# EYS Is a Protein Associated with the Ciliary Axoneme in Rods and Cones

**DOI:** 10.1371/journal.pone.0166397

**Published:** 2016-11-15

**Authors:** Giovanna Alfano, Przemyslaw M. Kruczek, Amna Z. Shah, Barbara Kramarz, Glen Jeffery, Andrew C. Zelhof, Shomi S. Bhattacharya

**Affiliations:** 1 Institute of Ophthalmology, UCL, London, EC1V 9EL, United Kingdom; 2 Department of Biology, Indiana University, 1001 East Third St., Bloomington, IN, 47405, United States of America; University of Massachusetts Medical School, UNITED STATES

## Abstract

**Purpose:**

Mutations in the *EYS* gene are a common cause of autosomal recessive retinitis pigmentosa (arRP), yet the role of the EYS protein in humans is presently unclear. The aim of this study was to investigate the isoform structure, expression and potential function of EYS in the mammalian retina in order to better understand its involvement in the pathogenesis of arRP.

**Methods:**

To achieve the objective, we examined the expression of mRNA transcripts of EYS isoforms in human tissues and cell lines by RT-PCR. We also investigated the localisation of EYS in cultured cells and retinal cryo-sections by confocal fluorescence microscopy and Western blot analysis.

**Results:**

RT-PCR analysis confirmed that EYS has at least four isoforms. In addition to the previously reported EYS isoforms 1 and 4, we present the experimental validation of two smaller variants referred to as EYS isoforms 2 and 3. All four isoforms are expressed in the human retina and Y79 cells and the short variants were additionally detected in the testis. Immunofluorescent confocal microscopy and Western blot analysis revealed that all EYS isoforms preferentially localise to the cytoplasm of Y79 and HeLa cells. Moreover, an enrichment of the endogenous protein was observed near the centrosomes in Y79 cells. Interestingly, EYS was observed at the ciliary axoneme in Y79 ciliated cells. In macaque retinal cryosections, EYS was found to localise in the region of the photoreceptor ciliary axoneme in both rods and cones as well as in the cytoplasm of the ganglion cells.

**Conclusion:**

The results obtained in this study lead us to speculate that, in photoreceptor cells, EYS could be a protein involved in maintaining the stability of the ciliary axoneme in both rods and cones. The variability of its isoform structure suggests that other roles are also possible and yet to be established.

## Introduction

Retinitis pigmentosa (RP, OMIM #268000) is a heterogeneous group of inherited retinopathies characterised by progressive degeneration of photoreceptor cells leading to visual impairment and eventually to complete blindness [1]. The worldwide prevalence of RP is about 1 in 3500–5000 with more than 1 million individuals diagnosed with the disease, which makes it the most common inherited photoreceptor degeneration [2]. Mutations in the *EYS* gene are the commonest cause of non-syndromic autosomal recessive retinitis pigmentosa (arRP; OMIM #602772) [3, 4]. The mutation prevalence ranges from 5% in the Dutch and Canadian populations [5] to 18–23.5% in the Japanese population [6], where mutations in *EYS* have emerged as the most frequent cause of inherited retinal dystrophies overall [7]. Of note, there has been a report of a Japanese individual diagnosed with cone-rod dystrophy caused by a compound heterozygous mutation in *EYS* [8]. The majority of patients harbouring mutations in *EYS* presents with a typical form of arRP. On average, it has been estimated that the visual field in patients with mutations in *EYS* begins to progressively deteriorate at around 30 years of age [9].

Spanning over 2 Mb of genomic DNA, *EYS* is one of the largest genes known to be expressed in the retina. Abd El-Aziz *et al*. reported *EYS* to be composed of 43 exons whereas the study conducted by Collin and co-workers revealed a similar gene structure with an additional exon between exon 41 and 42, making the predicted protein slightly larger consisting of 3165 amino acids. The alternative 42nd exon comprises 63 bp and lies just prior to the sequence encoding the fourth Laminin G-like domain [3, 4]. The initially described transcripts are currently annotated as EYS isoform 1 (3144 aa) and EYS isoform 4 (3165 aa) and both of them are predicted to encode a protein of identical domain structure. The EYS protein consists of a signal peptide followed by 21 epidermal growth factor (EGF) -like domains, putative coiled-coil domain and five Laminin G-like domains that are interspersed by EGF-like repeats. Furthermore, it has been suggested that the signal peptide and its cleavage site consensus sequence located in the N-terminal region of EYS may confer a secretory character to the protein or result in an intracellular or cytoplasmic localisation of the mature protein [3, 4, 10].

In the course of evolution, the expression of *EYS* was lost in several lineages of mammals, including rodents. However, EYS was discovered to be an orthologue of a *Drosophila melanogaster* protein called EYS/Spacemaker [3, 4]. It has been demonstrated that *Drosophila* EYS is an extracellular protein, which is a member of a network of interactions critical to the formation of the inter-rhabdomeral space (IRS) in the *D*. *melanogaster* ommatidium [11, 12] Zelhof *et al*. have also shown that *Drosophila* EYS interacts with Prominin during the IRS formation and a subsequent study suggested that this interaction may be conserved in humans and compromised in retinopathies [12, 13]. In another study, *Drosophila* EYS was found to be expressed in the mechanoreceptor neurons in *D*. *melanogaster* where it was shown to protect the neurons from environmental insult by preservation of the cell shape enforced by stiffening of the cell membrane [14].

In the present study, we examined the expression of EYS isoforms in human tissues and investigated their subcellular localisation patterns in cell-based models as well as retinal cryosections in order to gain insights into the function of EYS.

## Materials and Methods

### Expression studies

Total RNA was extracted from cell lines using the TRIzol extraction kit (Invitrogen) according to the manufacturer’s instructions. For expression studies, a panel of human total RNAs (Clontech) was used to prepare cDNA, using the QuantiTect® Reverse Transcription kit (Qiagen), according to the manufacturer’s instructions. RT-PCR was performed to amplify the coding sequence of the human *EYS* isoform 2 and 3 using GoTaq® Colorless Master Mix 2X (Promega) and transcript-specific primers (S1 Table).

The cDNA PCR products were sub-cloned in pcRII TOPO Vector (Invitrogen) and were analysed by direct sequencing. peGFP-C3-*EYS* and p3XFlag-*EYS* isoforms constructs were generated by amplification with GoTaq® Colorless Master Mix 2X (Promega) using *EYS* in pcRII TOPO Vector (Invitrogen) and primers with tails containing restriction enzyme sites. *EYS* isoforms were cloned at the *XhoI/KpnI* sites in peGFP-C3 (Clontech, 6082–1) and at *EcoRI/KpnI* sites in p3XFLAG-*myc*-CMV™-26 (Sigma, E6401). DNA sequencing was performed using a BigDye^TM^ Terminator v3.1 Cycle Sequencing Kit (Applied Biosystems, UK) and ABI PRISM^®^ 3730 Genetic Analyzer, according to the manufacturer’s protocols. The sequencing electropherograms were analysed using Lasergene DNASTAR^®^ software (DNASTAR, Inc). The reference sequences were obtained from Ensembl database [15]. Data regarding annotated *EYS* isoforms were obtained from Ensemble, GeneBank and UCSC genetic databases [15–17] and from UniProt protein database [18]. Predictions of protein domain structure were performed using ExPASy-Prosite protein prediction tool [19].

### Cell culture

Human HeLa epitheloid cervix carcinoma cells were grown at 37°C and 5% CO_2_ in DMEM supplemented with 10% FCS, penicillin (100 U/ml) and streptomycin (50 μg/ml). Human Y79 retinoblastoma cells were cultured in RPMI 1640 supplemented with 20% FCS, penicillin (100 U/ml) and streptomycin (50 μg/ml). Both cell lines were purchased from ATCC and the experiments were performed with cells of early passages.

To immobilise Y79 cells, they were seeded on glass coverslips coated with poly-L-lysine (Sigma-Aldrich, MO, USA) at concentration 2 μg/cm^2^. The coverslips were incubated with poly-L-lysine solution for 2 hours at 37°C. Next, they were rinsed with sterile water and the suspension of Y79 cells was added. For the purpose of cell attachment Y79 cells were suspended and seeded in complete DMEM media.

In order to enhance the growth of Y79 cells, they were treated with dibutyryl cyclin AMP (dbcAMP; Sigma-Aldrich, MO, USA) according to previously published protocols [20–22]. Briefly, the cells were seeded on poly-L-lysine coated coverslips and cultured in serum and antibiotic free DMEM medium supplemented with dbcAMP at the final concentration of 2 mM. The agent was re-administered every 2 days with media changes. After 5 days of treatment the cells were fixed and immunolabelled.

For transfection purposes, cells were seeded on glass coverslips at the density of 10^4^ per cm^2^. For Y79 cells, glass coverslips were pre-coated with poly-L-Lysine (Sigma-aldrich, MO, USA). The cells were grown for 12 h and transfected with 250–500 ng of DNA using Lipofectamine^®^ 2000 (Thermo Fisher Scientific, USA) according to the manufacturer’s instructions.

### Antibodies

Two custom made rabbit polyclonal anti-EYS antibodies whose epitopes were respectively against an N-terminal sequence [3] or a sequence in the central part of EYS isoform 1/4) were used for immunofluorescence (IF) at 1:50 (sections) or 1:300 (cells) and at 1:500 for Western blotting (WB), a commercially available goat polyclonal anti-EYS antibody (sc-167732) was used for immunofluorescence at 1:20 (cells). Mouse monoclonal anti-Acetylated-α-tubulin antibody (clone 6-11B-1, Sigma-Aldrich, USA) was used at 1:5000 for IF. Mouse monoclonal anti-α-tubulin antibody (clone DM1A; Sigma-Aldrich, USA) used at 1: 5000 for IF. Rabbit anti-CEP135 antibody (Abcam) was used at 1:100 for IF. Mouse monoclonal anti-β-dystroglycan antibody (Sigma-Aldrich, USA) was used at 1:1000 for WB. Mouse monoclonal anti-arrestin antibody (SantaCruz, USA) was used at 1:500 for IF. Chicken anti-RP1 antibody (gift from Dr Eric Pierce) was used at 1:100 for IF. Mouse anti-Rhodopsin (Abcam) was used at 1:10000 for IF. Goat anti-S-Opsin antibody (SantaCruz, USA) was used at 1:20 for IF. Mouse anti-FLAG antibody (clone F3165, Sigma-Aldrich, USA) was used at 1:10000 for IF and at 1:2000 for WB. Alexa Fluor 488 conjugated goat anti-rabbit and donkey anti-goat (Thermo Fisher Scientific, USA) and Cy3 conjugated goat anti-mouse, goat anti-chicken and donkey anti-rabbit (Jackson Immuno Research, USA) secondary antibodies were used at 1:400 dilution. HRP conjugated goat anti- mouse and goat anti-rabbit antibodies (Jackson Immuno Research, USA) were used for WB at 1:15 000 dilution.

### Immunocytochemistry

For the purpose of immunocytochemistry, cells were fixed for 20 minutes in 4% paraformaldehyde (PFA) in PBS. Next, they were rinsed with 20 mM glycine in PBS and incubated in the same solution for 15 minutes to quench free aldehyde groups of PFA. Coverslips were then blocked in blocking buffer (6% BSA, 0.3% Tween20, PBS) for 15 minutes and incubated with primary antibodies overnight at room temperature. This was followed by PBS washes and incubation with secondary antibodies for 45 minutes at room temperature in darkness. Primary and secondary antibodies were diluted in blocking buffer as described above. Negative controls were obtained by omitting the primary antibody. Cell nuclei were stained with 4’,6-diamidino-2-phenylindole at 1:2500 dilution (DAPI; Sigma-Aldrich, MO, USA) whereas F-actin filaments were visualised by staining with Alexa Fluor® 594 conjugated phalloidin at 1:400 dilution (Thermo Fisher Scientific, MA, USA). The coverslips were rinsed with PBS and mounted using DAKO Fluorescence Mounting Medium (Agilent Technologies, CA, USA). Confocal images were obtained using a ZEISS LSM 700 laser scanning confocal microscope (Carl Zeiss, Germany). Images were processed using ZEN (black edition) Image Browser and Adobe Photoshop CS5 (Adobe Systems, WA, USA).

### Immunohistochemistry

Immunohistochemical analysis was performed on adult monkey retina (*Macaca fascicularis*, 2 and 3 years old). The primate tissues came from a government facility (MRC facility at Porton Down) that had full approval for primate use both via local institutional ethical approval and national ethical and legislative regulation and under the UK Home Office Animals (Scientific Procedures) Act 1986. The animals lived in family groups with free access to food and water. However, the tissues obtained here were harvested from animals following their death. Primates were sedated with 0.1 mg/kg of Dormitol and 200mg/kg of ketamine given IM. They were bled out and humanly killed with 2.5ml Nembutal (50mg sodium pentobarbital per ml). The reason for the animals use was other than that reported in this study, but we were given access to the body specifically for collecting the eyes at post mortem immediately after death.

Briefly, the eyes were fixed in 4% PFA in PBS and cryoprotected with a sucrose gradient (10–30%). The eyes were then embedded in O.C.T. compound (VWR, UK) and cryosectioned at 5–10 μm. To retrieve antigens, the cryosections were treated with 0.01 M Citrate buffer at high temperature and then permeabilised with 0.3% Triton-X in PBS for 5 minutes. The sections were incubated in blocking buffer (5% donkey or goat serum, 6% BSA, 0.3% Tween20 in PBS) for 2 h at room temperature followed by incubation with primary antibodies for 18 h at room temperature and with secondary antibodies for 45 minutes at room temperature. The antibodies were diluted in blocking buffer at concentrations as described above. Negative controls were obtained by omitting the primary antibody. Peanut agglutinin (PNA) staining was performed by treatment with Rhodamine conjugated PNA (Vector Laboratories, UK) diluted at 1:100 for 1 h at room temperature. Cell nuclei were stained with DAPI at 1:5000 for 10 minutes at room temperature. Slides were mounted using DAKO Fluorescence Mounting Medium (Agilent Technologies, CA, USA). Confocal and DIC images were obtained using a ZEISS LSM 700 laser scanning confocal microscope (Carl Zeiss, Germany). Images were processed using ZEN (black edition) Image Browser and Adobe Photoshop CS5 (Adobe Systems, WA, USA).

### SDS-PAGE and Western blot analysis

Total cell lysates were prepared from HeLa and Y79 cells in RIPA buffer. Membrane proteins were isolated from Y79 cells using ProteoExtract® Native Membrane Protein Extraction Kit (Merk Millipore, Germany). Prior to SDS-PAGE and Western Blot analysis protein concentrations were determined using the BCA Protein Assay Kit (Merck Group, Germany). Protein samples were denatured in Laemmli Sample Buffer at 75°C for 10 minutes and loaded onto polyacrylamide gels for SDS-PAGE analysis (approximately 40 μg of sample were used per each input lane) followed by Western blotting. Mini-PROTEAN TGX Precast Gels (4–20%) and Trans-Blot Turbo Mini PVDF Transfer Packs were used. Membranes were blocked in 5% non-fat dried milk with 0.1% Tween20 in TBS. Primary and secondary antibodies were diluted in blocking buffer as described above. ECL solution (BioRad, CA, USA) was used for development and visualization was performed using the GelDoc XR+ System (BioRad, USA). The images were processed using the Image Lab software (BioRad, USA).

## Results

### EYS has four isoforms expressed in the retina

**Fig 1:**
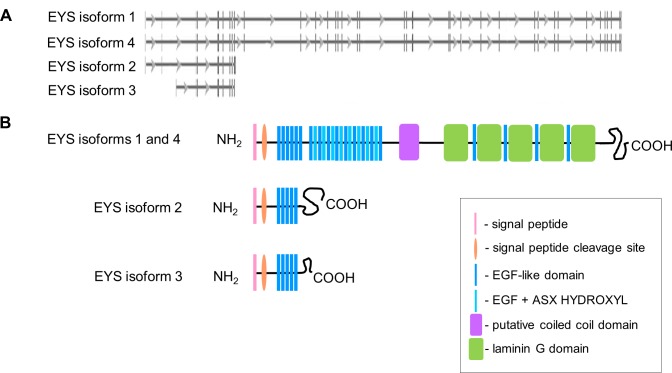
EYS has four isoforms annotated in genomic and protein databases: a summary of the genomic and protein structure of EYS isoforms. **(A)** A schematic view of EYS transcript variants. Perpendicular bars represent protein coding exons, horizontal lines represent introns and arrows indicate the transcriptional direction (data from NCBI Gene database; http://www.ncbi.nlm.nih.gov/gene/346007). **(B)** Predicted protein domain structure of EYS isoforms; domain order of EYS isoforms 1 and 4 is presented as previously described whereas the domain structure of EYS isoforms 2 and 3 was predicated using the Prosite ExPasy database.

EYS was previously reported to have two isoforms, both of which were predicted to encode retina-specific proteins [3, 4]. At the same time, online genomic and protein databases suggest the existence of further mRNA transcript variants: GenBank reports the existence of four EYS isoforms which is consistent with the data annotated in Ensembl and UCSC genome browsers as well as UniProt protein database. UniProt database, however, provides nomenclature of EYS isoforms that is different to the nomenclature provided in NCBI and UCSC databases. In this study, the nomenclature from the latter was followed and for clarity, details of EYS isoforms are summarised in Table 1 and Fig 1A.

**Table 1:** An overview of human EYS isoforms.

Isoform	cDNA	Protein [aa]	RefSeq (GenBank)	Variation (UniProt)
Isoform 1	10589	3144	NM_001142800.1, NP_001136272	2691–2711 aa: Missing
Isoform 2	5450	619	NM_001142801.1, NP_001136273	595–3165 aa: Missing; 590–594 aa: CSCSL → RILNTVIPHQIQQHIERFIQHDQVGFIVRI
Isoform 3	2168	594	NM_198283.1, NP_938024	595–3165 aa: Missing; 590–594 aa: CSCSL → YLCII
Isoform 4	10485	3165	NM_001292009.1, NP_001278938	canonical

The summary is based on collective data from GenBank and UniProt databases. NM numbers refer to transcript whereas NP numbers to protein variants. The amino acid alteration is based on data from UniProt.

Unlike the domain composition of EYS isoforms 1 and 4, the structure of EYS isoforms 2 and 3 had not been investigated before. Since the first 589 aa of all of the EYS isoforms are identical, we expected that EYS isoforms 2 and 3 also possess the N-terminal signal peptide and the run of EGF-like domains. For full verification of that hypothesis, we performed sequence scanning using the ExPASy-Prosite protein prediction tool. This revealed that both EYS isoforms 2 and 3 are composed of five EGF-like domains, with a variable length and amino acid composition of the C-terminal ends of the protein (Fig 1B).

**Fig 2:**
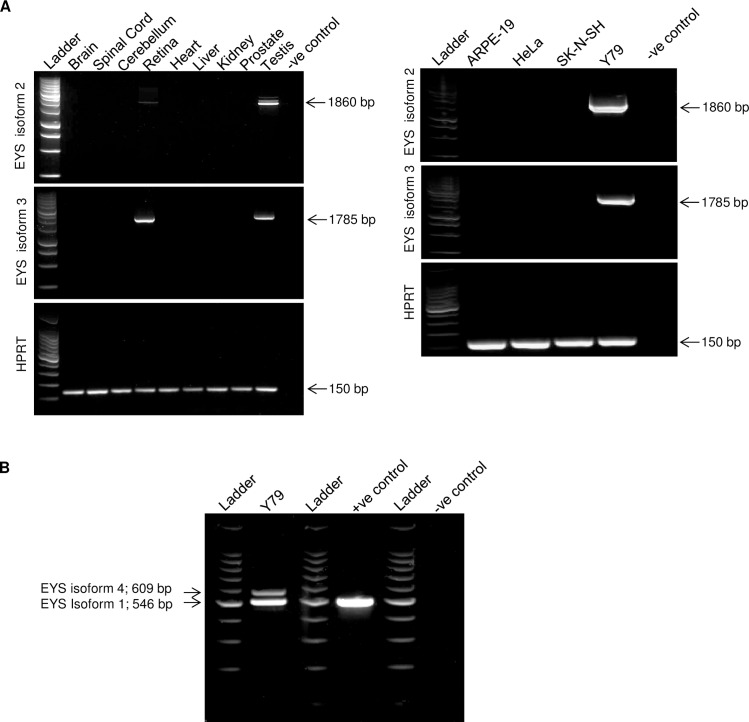
EYS isoforms are expressed in the retina, testis and Y79 retinoblastoma cell line: RT-PCR analysis of mRNA expression of EYS isoforms in human tissues and cell lines. **(A)** mRNA transcripts of EYS isoforms 2 and 3 are present in the cDNA from the human retina, testis and Y79 cell line. A fragment of the *HPRT* gene was used as a quantitative control and a reaction lacking template cDNA was used as a negative control (-ve control). **(B)** EYS isoform 4 is expressed in Y79 cell line as confirmed by amplification of EYS isoform 4-specific exon (upper band). In the positive control (+ve control), pcDNA3.1-EYS isoform 1 plasmid was used as template whereas a reaction lacking template cDNA was used as a negative control (-ve control).

Next, we experimentally validated expression of EYS isoforms 2 and 3 by performing RT-PCR analysis in a panel of human tissue and cell line derived cDNA samples, using isoform specific primers that encompass the full coding sequences of the analysed transcripts (S1 Table). According to our analysis, both EYS isoforms 2 and 3 are expressed in the retina, testis and Y79 cell line (Fig 2A). Of note, EYS isoform 3 is more abundantly expressed in the retina as indicated by RT-PCR assay, whereas EYS isoforms 2 displayed more than one band in the testis lane suggesting that there may be more isoforms present in that organ.

According to previously published data [3] and our results, Y79 cells are the only cells known to express EYS isoforms 1, 2 and 3; however, the expression of EYS isoform 4 had not been verified in this cell line. We, therefore, addressed this issue by performing RT-PCR analysis using primers capable of amplifying across the 42nd exon which is specific to EYS isoform 4. As shown in Fig 2B, two bands were detected: the higher band of 609 bp indicates expression of EYS isoform 4 while the amplicon of 546 bp represents expression of EYS isoform 1. This confirmed that EYS isoform 4 is also expressed in Y79 cells.

In summary, all of four transcript variants for EYS are expressed in the human retina and Y79 cell line, EYS isoforms 2 and 3 are also detected in the testis. Moreover, our data suggest that Y79 cell line is the only cell line available for investigation of endogenous expression patterns of EYS proteins.

### EYS localises to the cytoplasm, centrosomes and ciliary axoneme in Y79 cell line

To examine the endogenous expression pattern of EYS protein we used Y79 retinoblastoma cells. Y79 cells grow in suspension as multicellular grape-like aggregates, however, it has been reported that they are capable of growing as a monolayer on poly-L-lysine coated surfaces and that they can acquire neuron-like morphology when exposed to agents such as dbcAMP or insulin [20–22]. In this study, we exploited these discoveries and cultured Y79 cells on poly-L-lysine coated glass coverslips. For a subset of experiments we used cells treated with dbcAMP to enhance cell growth. Analysis of EYS localisation was performed using all of the three antibodies available in our study and produced similar results.

**Fig 3:**
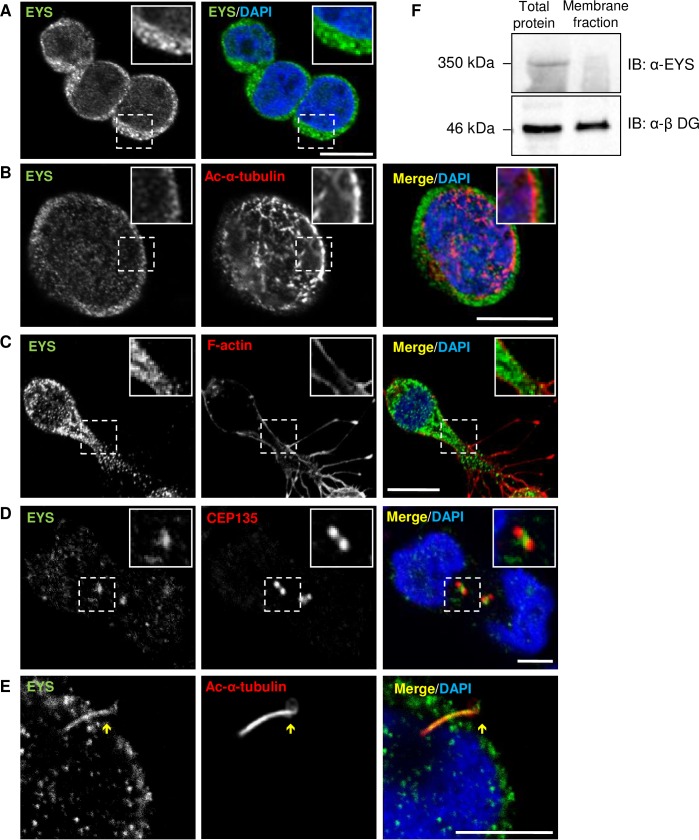
EYS localises to the cell cytoplasm, centrosomes and ciliary axoneme: immunofluorescent localisation and Western blot analysis in Y79 cells. **(A)** Immunofluorescent localisation of EYS (green) in a cluster of Y79 cells. **(B)** Co-labelling of EYS (green) with Acetylated-α-tubulin (Ac- α-tubulin; red). **(C)** Co-labelling of EYS (green) and F-actin (red) in dbcAMP treated Y79 cells. **(D)** Co-labelling of EYS (green) and CEP135 (red). (**E**) High magnification showing co-labelling of EYS (green) with Ac-α-tubulin (red) in a ciliated cell. The zoomed inserts represent the areas demarcated by the dashed boxes; the yellow arrows indicate the cilium. Cell nuclei are stained with DAPI (blue). Scale bars: 10 μm. **(F)** Western blot analysis of the presence of EYS in total protein extract versus membrane protein fraction obtained from Y79 cells. β-dystroglycan (β-DG) was used as an internal control and loading was optimised by BCA assay – 40 μg of protein were loaded in each well.

In Y79 cells, immunofluorescently labelled EYS was observed as a speckled signal forming a ring around the nucleus suggesting that it is mostly concentrated in the cytoplasm (Fig 3A); Texas Red-X conjugated Wheat Germ agglutinin (WGA) was used to visualise the cell membrane (S1 Fig). To visualise cytoskeletal structures, Acetylated-α-tubulin and α-tubulin were used as markers and the experiments showed that EYS, to some extent, could also associate with the cell cortex (Fig 3B and data not shown). This was in particular indicated by EYS forming a ring around the structures highlighted by anti-Acetylated-α-tubulin antibody and a similar pattern was seen in cells labelled anti-α-tubulin antibody.

Since Y79 cells are relatively small and round in shape, it was impracticable to assess the level of potential overlap of EYS signal between the cytoplasm and the cell membrane. To address this issue, Y79 cells were treated with dbcAMP to obtain larger cell bodies and elongated membrane protrusions. Subsequent co-labelling of EYS and F-actin filaments revealed absence of signal overlap, suggesting that EYS preferentially localises to the cytoplasm and indicate an unlikely association with the cell membrane (Fig 3C). Negative controls for EYS immunostainings were obtained by omitting the primary antibody (S2 Fig). To reinforce our observations, we performed Western blot analysis to compare protein extracts from total cell lysate and the membrane protein fraction. As expected, EYS could only be detected in the total cell lysate and was absent in the membrane fraction, which confirms that it is not directly associated with the cell membrane (Fig 3F).

Since EYS was consistently detected as speckled rather than diffused signal, we also examined potential co-localisation of EYS with other cellular organelles. Intriguingly, enrichment of the fluorescence was observed in the areas of centrosomes, which were highlighted by immunolabelling of CEP135 protein (Fig 3D and S3 Fig). The centrosome serves as a microtubule organising centre, which suggests that EYS may be involved in the organisation of structures such as the primary cilium. Next, we performed co-labelling of EYS and Acetylated-α-tubulin, and interestingly, EYS was observed at the ciliary axoneme in Y79 ciliated cells (Fig 3E and S4 Fig).

### Overexpressed EYS isoform 1 localises to the cytoplasm of cultured cells

**Fig 4:**
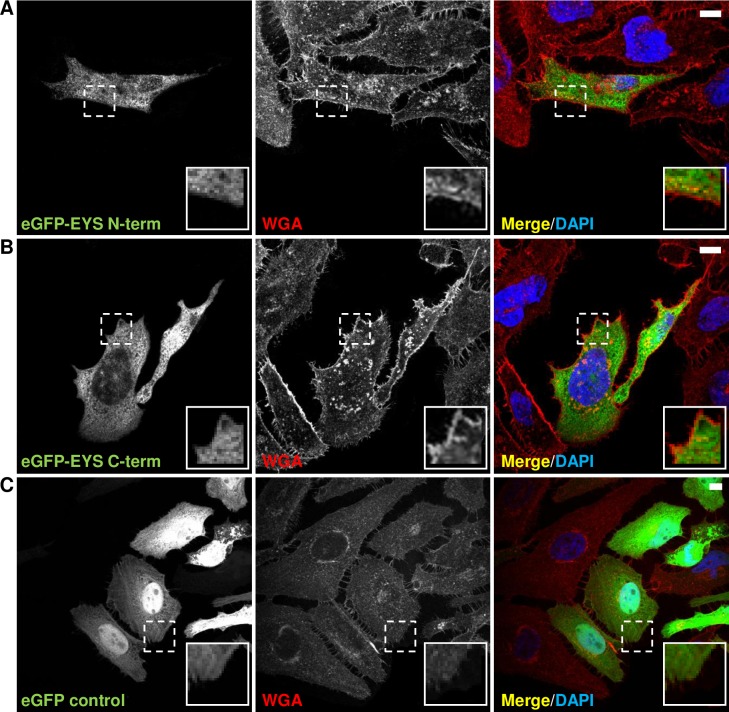
eGFP tagged fragments of EYS isoform 1 localise to the cell cytoplasm: immunocytochemical analysis in HeLa cells. **(A)** Localisation of the eGFP tagged N-terminal fragment of EYS isoforms 1 (1-1635 aa; eGFP-EYS N-term) in cells stained with WGA (red). **(B)** Localisation of the eGFP tagged C-terminal fragment (1880–3144 aa; eGFP-EYS C-term) in cells stained with WGA (red). **(C)** Localisation of eGFP tag in cells stained with WGA–eGFP tag-only control. The zoomed inserts represent the areas demarcated by the dashed boxes. Cell nuclei are stained with DAPI (blue). Scale bars: 10 μm.

To provide further insights into the localisation of EYS in cell lines, we analyzed the overexpressed protein isoforms. To begin with we attempted to express isoform 1, however, transfection efficiency was significantly hampered by the large size of the cDNA sequences (9435 bp). To circumvent this issue, we split the sequence of EYS isoform 1 in two parts and cloned each with an eGFP N-terminal tag. The N-terminal fragment comprised 1-1635 aa and encoded the run of 21 EGF-like domains and the coiled coil domain whereas the C-terminal fragment was composed of 1880–3144 aa and encoded the run of five LamG-like domains separated by EGF-like domains. Analysis was performed in HeLa cells because of their higher transfection efficiency compared to other cell lines available to us. It should be noted that recent findings described presence of cilia on HeLa and other cancer cell lines with a percentage higher than expected [23] suggesting that these cells can be a suitable system also for localisation studies involving proteins potentially associated with the primary cilium. The EYS fragments were overexpressed in cells, which were then stained with Texas Red-X conjugated Wheat Germ agglutinin (WGA) to visualise the cell membrane. Both of the tagged fragments of EYS isoform 1 localised to the cytoplasm with a clear absence of EYS signal from the cell membrane (Fig 4). Co-labelling of eGFP EYS fragments and CEP135 did not reveal localisation of EYS at the centrosomes, suggesting that either expression of full-length cDNA is required for localisation to these organelles or other isoforms could account for the centrosomal localisation of the endogenous EYS protein. Constructs of EYS isoform 4 were not available in the present study.

### EYS isoforms 2 and 3 reside in the cytoplasm and may form dimers

**Fig 5:**
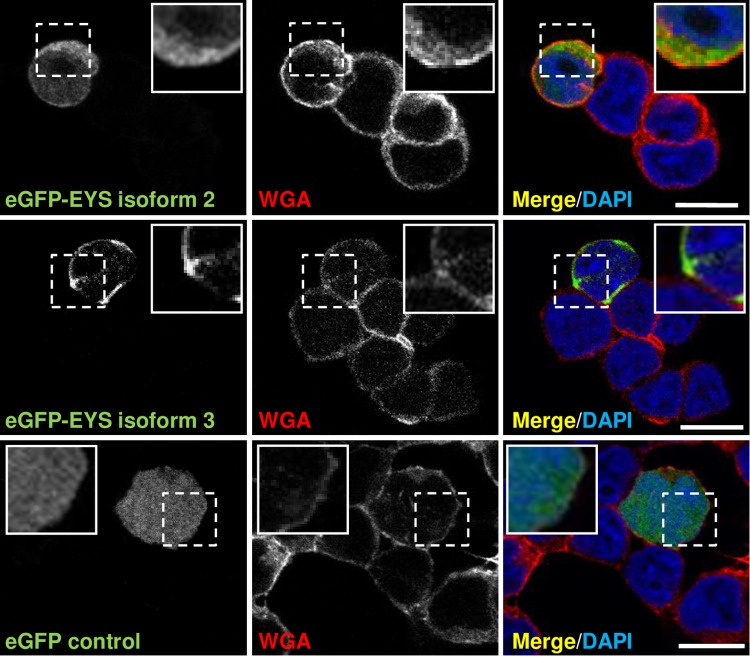
eGFP tagged EYS isoforms 2 and 3 localise to the cell cytoplasm: immunocytochemical analysis in Y79 cells. eGFP empty vector was used as a control and Texas Red-X conjugated WGA was used to stain the cell membrane. The zoomed inserts represent the areas demarcated by the dashed boxes. Cell nuclei are stained with DAPI (blue). Scale bars: 10 μm.

To examine the subcellular localisation of EYS isoforms 2 and 3, we tagged the proteins with N-terminal eGFP tag and overexpressed them in Y79 and HeLa cells. In Y79 cells, both EYS isoforms 2 and 3 were observed to form rings around the nucleus and no apparent overlap with WGA was observed, suggesting that they are concentrated in the cell cytoplasm (Fig 5).

**Fig 6:**
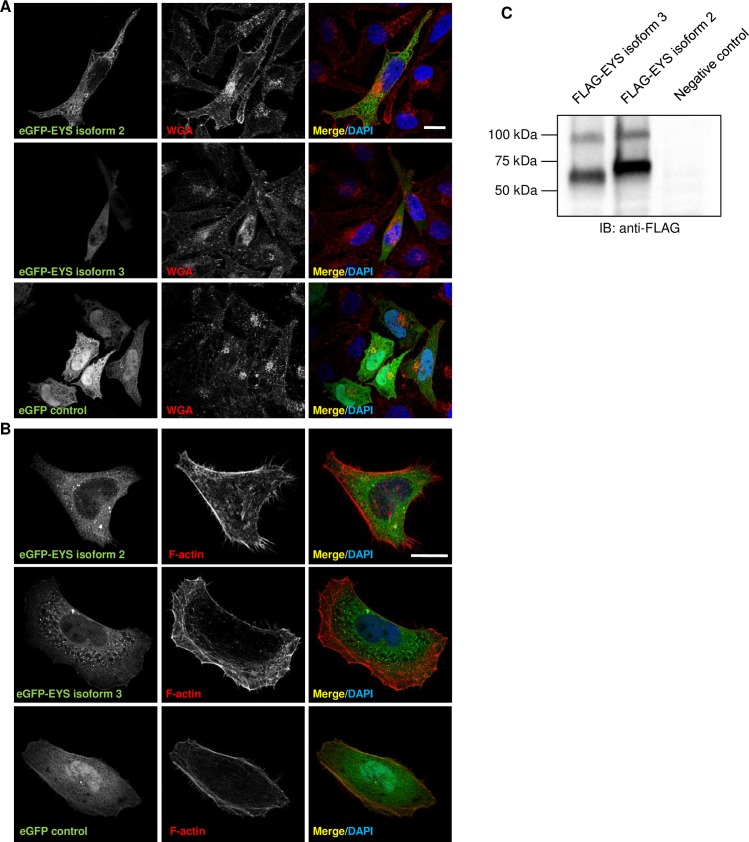
eGFP tagged EYS isoforms 2 and 3 localise to the cell cytoplasm and may form dimers: immunocytochemical and Western blot analysis in HeLa cells. **(A)** Localisation of the eGFP tagged EYS isoforms 2 and 3 in cells stained with WGA (red). **(B)** Localisation of the eGFP tagged EYS isoforms 2 and 3 in cells stained with phalloidin (red). eGFP empty vector was used as a control. The zoomed inserts represent the areas demarcated by the dashed boxes. Cell nuclei are stained with DAPI (blue). Scale bars: 10 μm. **(C)** Western blot analysis of FLAG tagged EYS isoforms 2 and 3. Protein extract from untransfected HeLa cells was used as a negative control. Loading was optimised by BCA assay – 40 μg of protein were loaded to each well.

Taking into account the drawbacks of using the Y79 cell line, the eGFP tagged constructs were transfected into HeLa cells in order to characterise the expression patterns with more accuracy. As expected, the eGFP tagged proteins were observed to localise to the cell cytoplasm in the transfected HeLa cells (Fig 6A and 6B). Analysis performed in SK-N-SH led to comparable results (data not shown). When imaging the transfected cells, we could occasionally observe speckles of more intense fluorescence. These, however, did not localise near the centrosomes, but were confirmed to co-localise with ubiquitin. This strongly suggests they were protein aggregates going through the clearance system of the cell and not associated with any particular organelle (S5 Fig).

Furthermore, we made an intriguing observation when separating FLAG tagged EYS isoforms 2 and 3 by SDS-PAGE. In addition to bands corresponding to the expected size of the isoforms (FLAG-EYS isoform 2 is 72 kDa whereas FLAG-EYS isoform 3 is 69 kDa), we could consistently detect upper bands at around 100 kDa (Fig 6C). This suggests that EYS isoforms 2 and 3 may be capable of forming dimers or other homocomplexes. Overall, our results suggest that EYS isoforms 2 and 3 preferentially localise to the cytoplasm of cultured cells and may be capable of forming higher order protein assemblies.

### EYS localises to the ciliary axoneme in rods and cones

**Fig 7:**
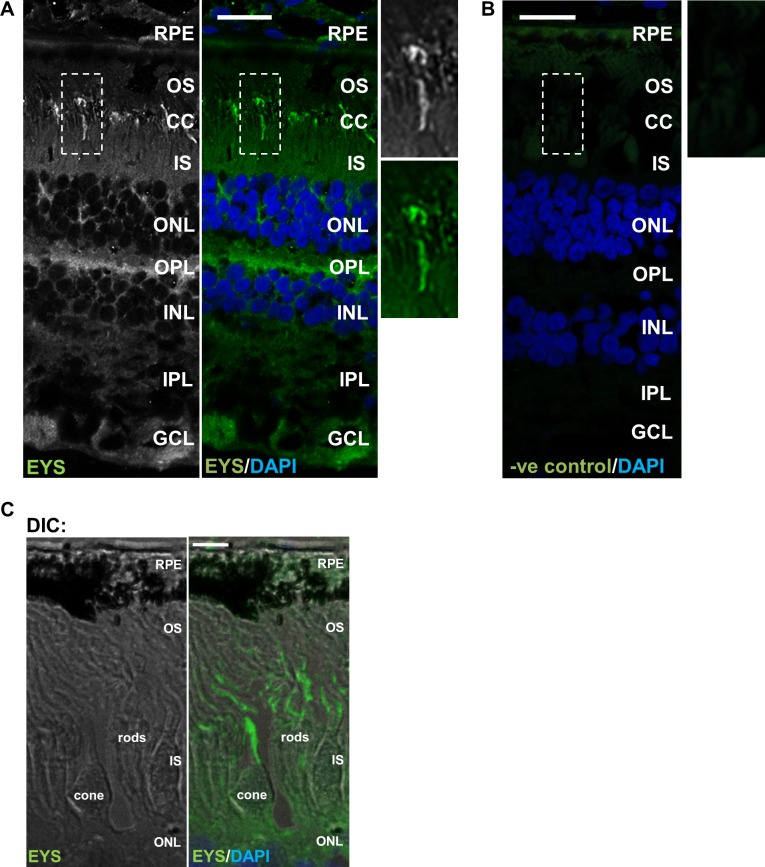
Fluorescently labelled EYS is detected as a whip-like signal in the outer retina: immunohistochemical analysis in macaque retinal cryosections. **(A)** Fluorescent localisation of EYS (green) in macaque retinal cryosections. The zoomed images represent the areas demarcated by dashed boxes. **(B)** The negative control (-ve control) was obtained by omitting the primary antibody. **(C)** The DIC (differential interference contrast) image illustrates the cytoarchitecture of the analysed photoreceptor cells. The layers of the retina are labelled as follows: RPE—retinal pigment epithelium, OS–outer segment, CC–connecting cilum, IS–inner segment, ONL–outer nuclear layer, OPL–outer plexiform layer, INL–inner nuclear later, IPL–inner plexiform layer, GCL–ganglion cell layer. Cell nuclei are labelled with DAPI (blue). Scale bars: 20 μm.

In previously published studies, the localisation of EYS in the retina was examined in the porcine tissue and it was demonstrated that the protein localises to the photoreceptor outer segments [3]. In this study, we took forward the immunohistochemical analysis of the localisation of EYS and used the retina from adult crab-eating macaques (*Macaca fascicularis*), a species whose retinal structure is closely related to that found in humans. Of note, immunostainings were performed using a custom made antibody (epitope corresponding to a sequence in the central part of EYS isoform 1 and 4) conserved in *Macaca fascicularis* as assessed by Blast sequence alignement. In single staining experiments, EYS was detected as a whip-like signal concentrated in the area corresponding to the connecting cilium and the base of the photoreceptor outer segments. There was also some signal detected in the outer plexiform and ganglion cell layers (Fig 7).

**Fig 8:**
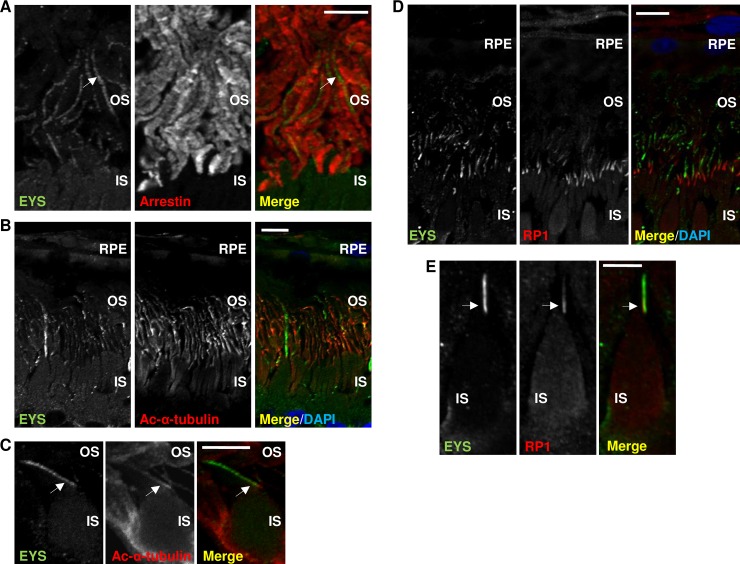
Fluorescently labelled EYS overlaps with markers of the outer segment and the ciliary axoneme: immunohistochemical analysis in macaque retinal cryosections. **(A)** EYS (green) overlaps with Arrestin (red) in the photoreceptor outer segments, the white arrows indicate an exemplary photoreceptor outer segment. **(B)** EYS (green) partially overlaps with Acetylated α-tubulin (Ac-α-tubulin; red). **(C)** A high magnification image demonstrating the overlap of EYS (green) and Ac-α-tubulin (red) in a single cell, the white arrows indicate the ciliary axoneme. **(D)** EYS (green) partially overlaps with RP1 (red). **(E)** A high magnification image demonstrating the overlap of EYS (green) and RP1 (red) in a single cell, the white arrows indicate the ciliary axoneme. The layers of the retina are labelled as follows: RPE—retinal pigment epithelium, OS–outer segment, IS–inner segment. Cell nuclei are labelled with DAPI (blue). Scale bars: 20 μm.

The localisation of EYS to the photoreceptor outer segments was ascertained by co-staining with Arrestin, an outer segment specific protein present in light adapted rod and cone photoreceptors (Fig 8A). To verify the presence of EYS in the connecting cilium we performed additional immunostainings with Acetylated-α-tubulin, which is a component of the ciliary axoneme, and RP1, which is known to associate with the photoreceptor ciliary axoneme. Again, EYS was detected as a whip-like signal and some overlap with Acetylated-α-tubulin was observed (Fig 8B and 8C). This was further supported by co-labelling of EYS and RP1 which occurred to partially overlap (Fig 8D and 8E). These results suggest that EYS is a component of or a protein associated with the photoreceptor ciliary axoneme, where it could participate in the maintenance of the photoreceptor outer segments.

**Fig 9:**
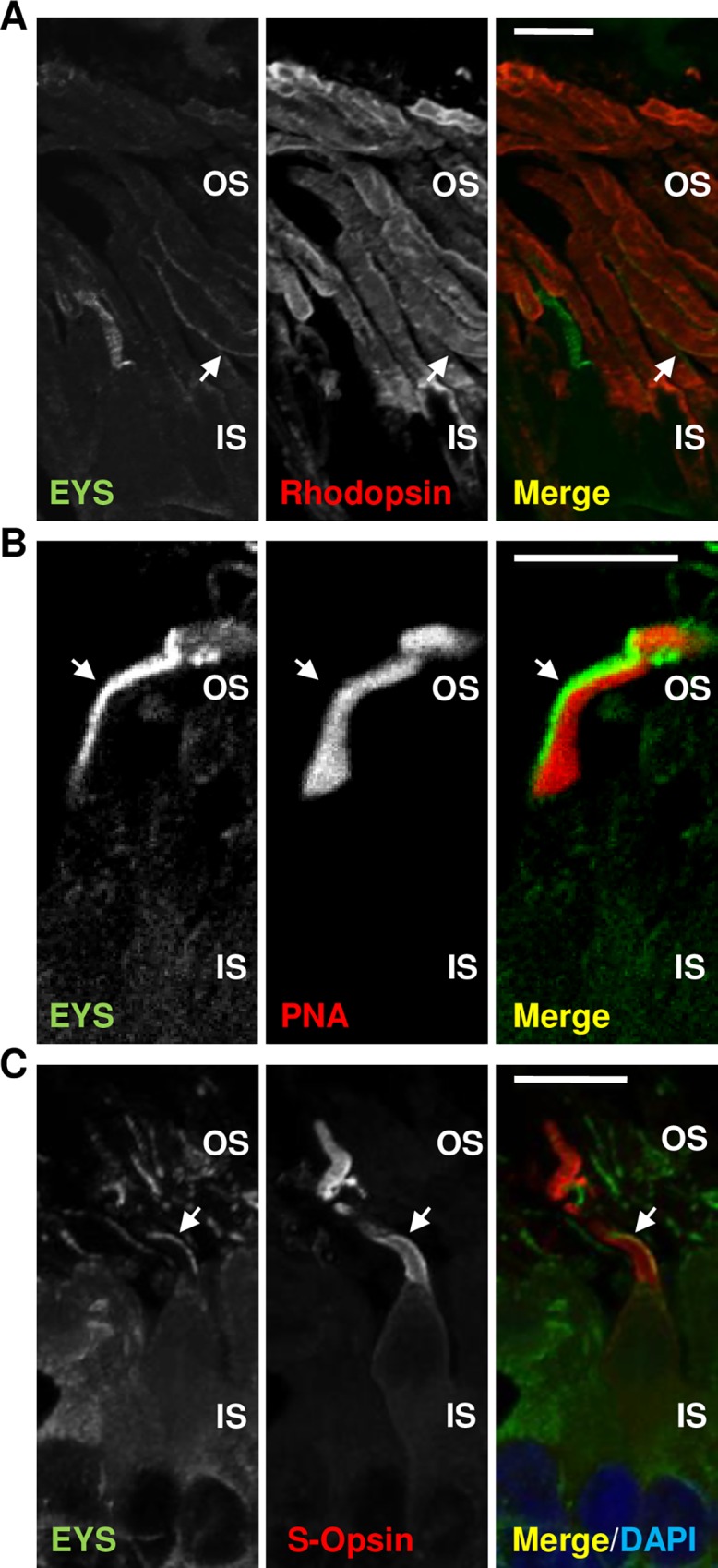
EYS is expressed in rods and cones: immunohistochemical analysis in macaque retinal cryosections. **(A)** EYS (green) is concentrated on one side of the rod outer segments labelled with Rhodopsin (red). The white arrows indicate the analysed rod outer segment. **(B)** EYS (green) concentrates unilaterally in the cone outer segment highlighted by PNA staining (red). The white arrows indicate the analysed cone outer segment. **(C)** EYS (green) concentrates unilaterally in the cone outer segment highlighted by S-opsin (red). The white arrows indicate the analysed cone outer segment. The layers of the retina are labelled as follows: OS–outer segment, IS–inner segment. Scale bars: 20 μm.

Mutations in *EYS* have been commonly found in individuals suffering from arRP; nonetheless, one case of cone-rod dystrophy caused by a compound heterozygous mutation in *EYS* has been reported [8]. We addressed the presence of EYS in rods and cones by performing co-stainings with markers specific to each type of photoreceptors. As expected, we detected EYS as unilaterally concentrated whip-like signal present in rods that were highlighted by labelling of Rhodopsin (Fig 9A). To verify the presence of EYS in cones, we used PNA, which specifically binds to the cone associated matrix proteins (Fig 9B), and S-opsin, which is a protein specifically expressed in S-cones (Fig 9C). Both experiments confirmed that EYS is also expressed in cones and it is characteristically concentrated to one side of the outer segments, which most likely corresponds to the ciliary axoneme according to our observations.

Together, our data indicate that EYS is a protein associated with the ciliary axoneme and it is expressed in both rods and cones, where it could be involved in the maintenance of the photoreceptor outer segments. The expression and function of EYS in other layers of the retina is yet to be investigated.

## Discussion

Identification of physiological functions of retinopathy associated proteins is vital for the development of potential therapeutic approaches. In this study, we present characterisation of the isoform structure and expression pattern of EYS, which provides novel insights into the pathophysiology of arRP caused by mutations in the *EYS* gene.

The analysis of isoform structure revealed that EYS has multiple variants and four are expressed in the retina. Alternative splicing is a dynamically regulated process that contributes to the proteomic diversity and it is especially common for genes expressed in the nervous system, where the synthesis of protein variants determines properties of different types of neurons [24, 25]. It has been estimated that 15% of disease causing point-mutations affect pre-mRNA splicing and impaired splicing is also known to contribute to retinal degeneration [26]. In addition to the previously discovered EYS isoforms 1 and 4, we have now confirmed the existence of two shorter splicing variants, EYS isoforms 2 and 3, which are also expressed in the testis. The testicular expression of EYS isoforms 2 and 3 is yet to be investigated; however, a testis related phenotype has not been described in males carrying mutations in *EYS*. Interestingly, mutation in EYS are frequent and there is no mutation hot-spot in the sequence of the gene and to date there are disease causing mutations in EYS that would affect the common sequence in all four isoforms investigated in this study.Nonetheless, the involvement of each of the isoforms in the pathogenesis of arRP remains to be established. Differences in the domain structure of EYS isoforms are striking and this variation could confer multiple functions on the proteins that may be differentially expressed depending on the developmental and/or physiological condition of an organism. This means that the analysis of the disease mechanisms related to each isoform will be critical in fully understanding the physiological role of EYS and disease progression.

To provide further insight into the physiological role of EYS, we first examined its localisation in cultured cells. We found that EYS preferentially localises to the cell cytoplasm with enrichment near the centrosomes. Interestingly, EYS was seen localising to the ciliary axoneme in Y79 cells, which suggest that EYS could participate in the organisation/function of microtubule structures such as the primary cilium. However, EYS isoforms 2 and 3 were observed only in the cytoplasm, which implies that their physiological role may be different to their full-length counterparts. Furthermore, EYS isoforms 2 and 3 were found to always separate as two bands on Westerns demonstrating that they may form dimers or other higher order protein assemblies. Taken together, these data suggest that the variable length and domain structure of EYS isoforms may confer differences in their potential function. Moreover, our results along with literature data, suggest that regardless of similar domain structure, in photoreceptors, *Drosophila* EYS and its human orthologue may undergo species specific post-translational modification, targeting the protein towards its final localisation. Similarly we may hypothesise that the signal peptide is not cleaved in the human leading to EYS localisation in the intracellular compartment whereas in *Drosophila* it appears to be extracellular. The analysis of the subcellular localisation of EYS in the macaque retina demonstrated that it is present in both rods and cones, and was detected as a unilaterally concentrated whip-like signal that extends from the connecting cilium towards the tip of the outer segments. Since mutations in *EYS* are implicated in arRP, the presence of EYS in cone cells was unexpected. In light of the report of a case of cone-rod dystrophy caused by mutations in EYS [8], this finding leads to a conclusion that EYS is also vital for the proper functioning of cones. The observed partial overlap of EYS and components of the ciliary axoneme provides further support to the hypothesis that EYS can be involved in structural organisation of the outer segments. Our results seen in context with previously published data lead us to speculate that EYS could be a protein involved in maintaining the stability of the photoreceptor ciliary axoneme; however, the variability of its isoform structure suggests that other functions are also plausible. This hypothesis is consistent with the previously published studies performed in *D*. *melanogaster*. For example, in Drosophila mechanosensory neurons, not only does EYS localise to the surface of the neuron, it is also tightly associated with the cilium [14]. Whereas, the apparent difference in localisation patterns in vertebrate and Drosophila photoreceptors may simply be attributed to the structural and organisational differences between the rhabdomeric and ciliated photoreceptors. Notably, the domain structure of the *D*. *melanogaster* and human orthologue is very similar and thus despite different subcellular localisation, they could play an equivalent role in maintaining the structural integrity of photoreceptors.

## Supporting Information

S1 Fig Endogenous EYS localises to the cell cytoplasm: colabelling of EYS and WGA in Y79 cells. Immunofluorescent localisation of EYS (green) in a cluster of round cells labelled with WGA (red). Cell nuclei are labelled with DAPI (blue). n–nucleus, c–cytoplasm. Scale bars: 10 μm.
(TIF)

S2 Fig **Endogenous EYS localises to the cell cytoplasm (A)** Immunofluorescent localisation of EYS (green) in a cluster of Y79 cells labelled with phalloidin (red). **(B)** The negative control (-ve control) was obtained by omitting the primary antibody. Cell nuclei are labelled with DAPI (blue). Scale bars: 10 μm.
(TIF)

S3 Fig EYS localises to the cell cytoplasm and is enriched near the centrosomes throughout the cell cycle: immunfluorescent localisation of EYS (green) and CEP135 (red) in Y79 cells. Cell nuclei are labelled with DAPI (blue). Scale bars: 10 μm.
(TIF)

S4 Fig EYS localises to the ciliary axoneme in Y79 cells: immunofluorescent localisation of EYS (green) and Acetylated-α-tubulin (red). The ciliary axoneme is indicated by the arrows. Cell nuclei are labelled with DAPI (blue). Scale bars: 10 μm.
(TIF)

S5 Fig EYS isoforms 2 and 3 localise to the cell cytoplasm and the stronger speckles of signal co-localise with ubiquitin: immunofluorescent localisation of EYS isoforms 2 and 3 (green) and Ubiquitin (red) in HeLa cells. The white arrows indicate the speckles of protein going through the ubiquitin-proteasomal protein clearance system of the cell. Cell nuclei are labelled with DAPI (blue). Scale bars: 10 μm.
(TIF)

S1 Table A summary of primer sequences used for RT-PCR analysis of transcript levels of EYS isoforms in the human retina and Y79 cell line. (DOC)
