# Supplementary figures and images for: EYS Is a Protein Associated with the Ciliary Axoneme in Rods and Cones
Source: PLoS One. 2016 Nov 15;11(11):e0166397. doi: 10.1371/journal.pone.0166397 (PMC5112921; doi:10.1371/journal.pone.0166397)

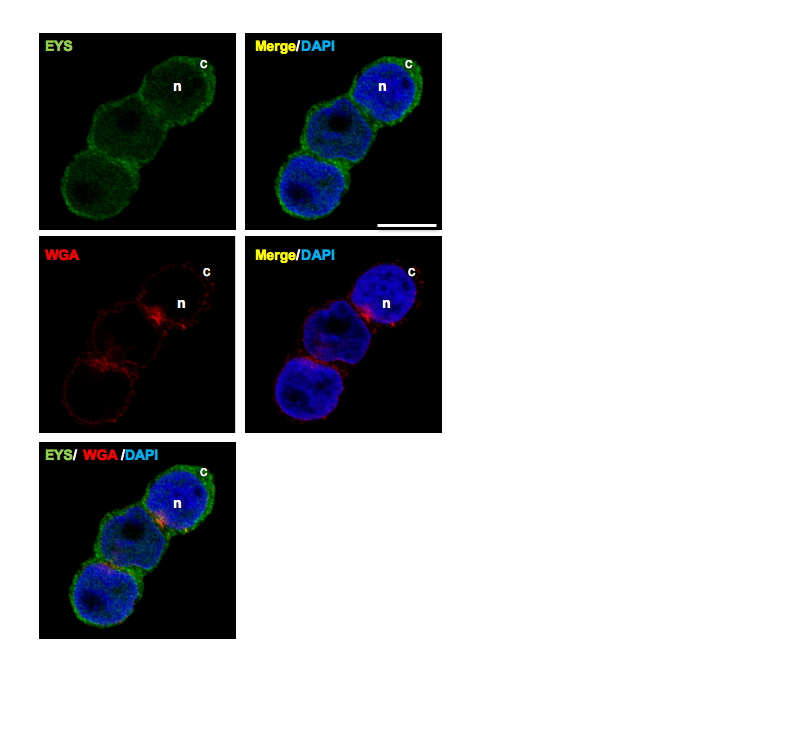

Supplement: S1 Fig — Immunofluorescent localisation of EYS (green) in a cluster of round cells labelled with WGA (red). Cell nuclei are labelled with DAPI (blue). n–nucleus, c–cytoplasm. Scale bars: 10 μm. (TIF) [file pone.0166397.s001.tif]

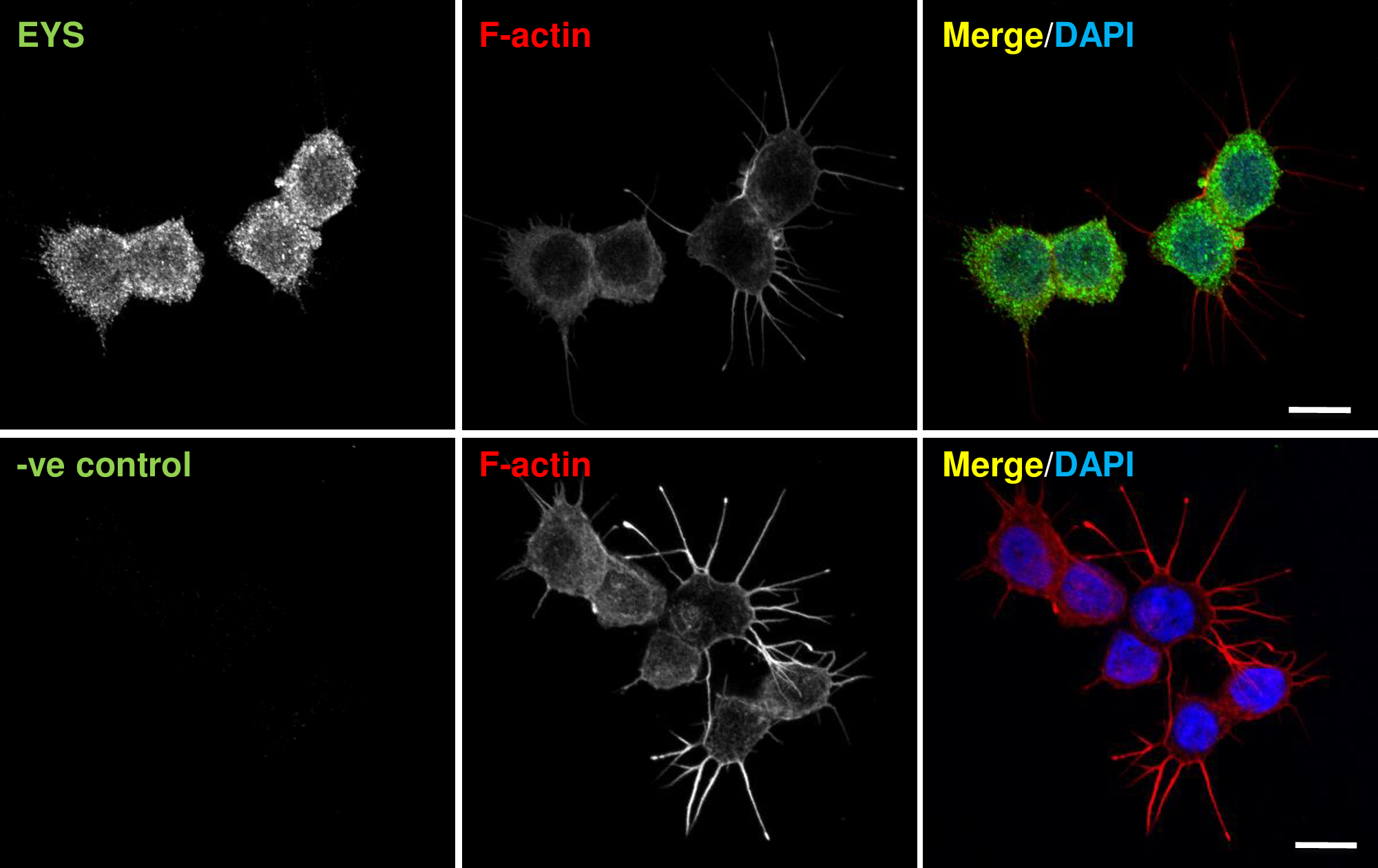

Supplement: S2 Fig — Endogenous EYS localises to the cell cytoplasm (A) Immunofluorescent localisation of EYS (green) in a cluster of Y79 cells labelled with phalloidin (red). (B) The negative control (-ve control) was obtained by omitting the primary antibody. Cell nuclei are labelled with DAPI (blue). Scale bars: 10 μm. (TIF) [file pone.0166397.s002.tif]

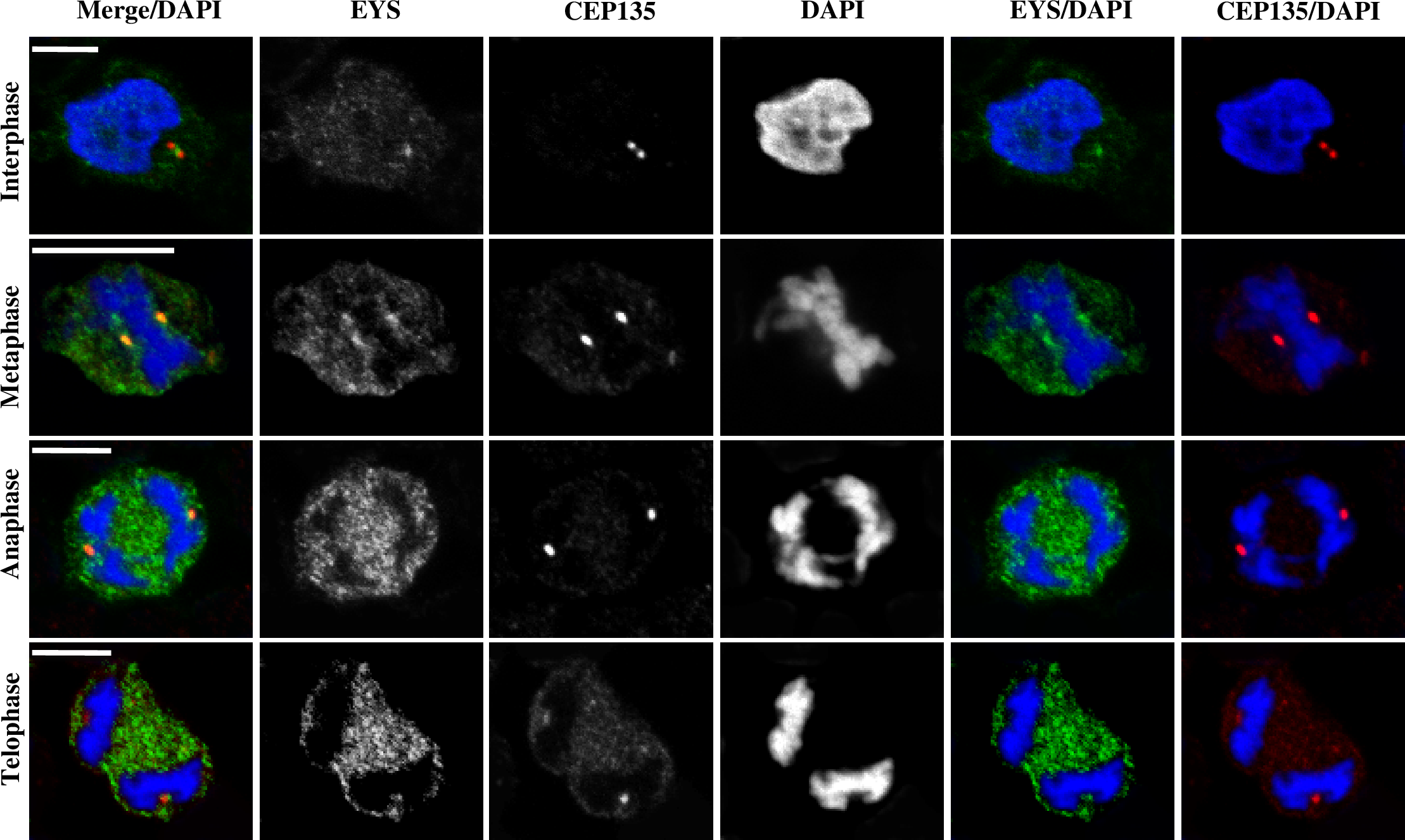

Supplement: S3 Fig — Cell nuclei are labelled with DAPI (blue). Scale bars: 10 μm. (TIF) [file pone.0166397.s003.tif]

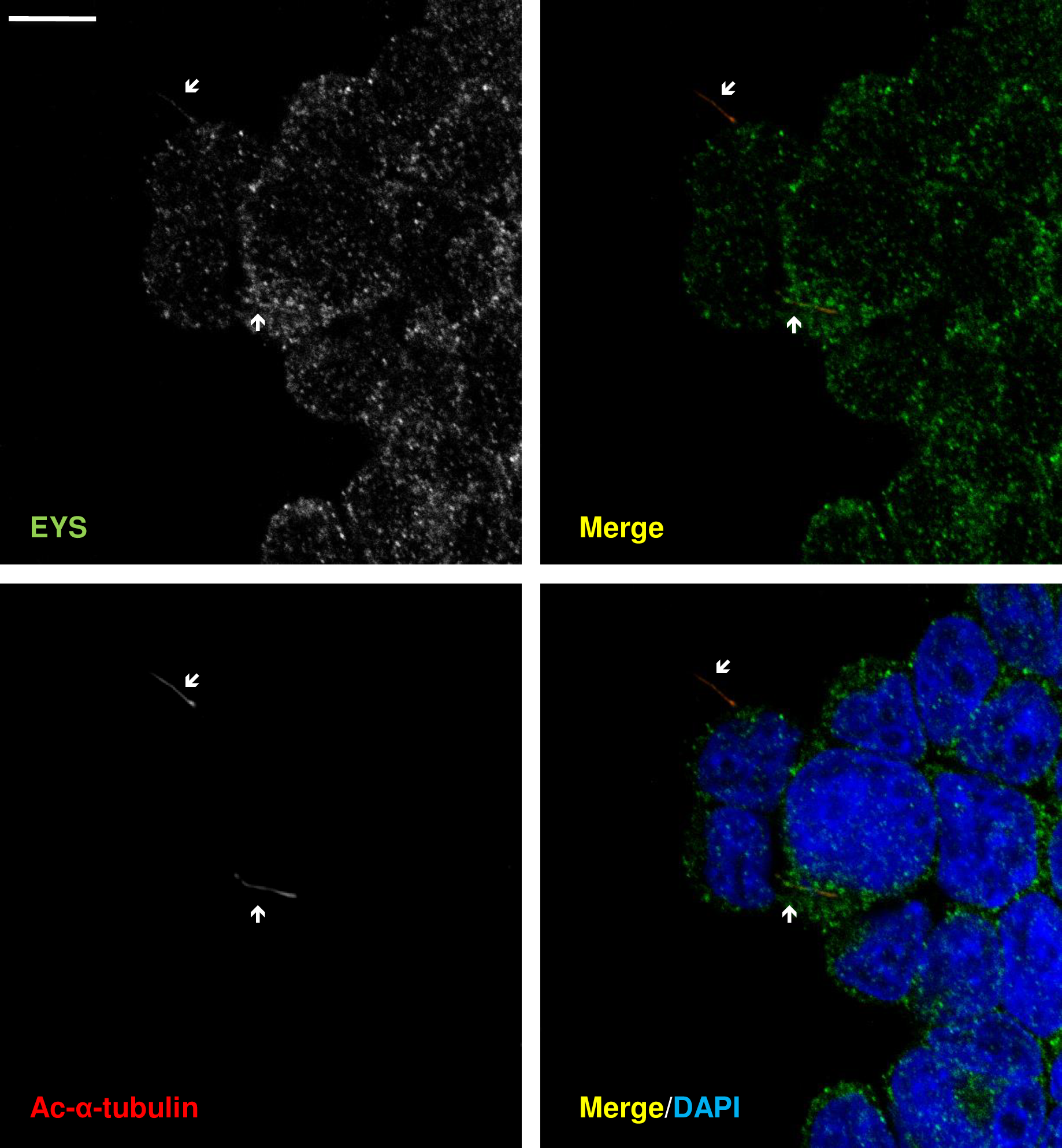

Supplement: S4 Fig — The ciliary axoneme is indicated by the arrows. Cell nuclei are labelled with DAPI (blue). Scale bars: 10 μm. (TIF) [file pone.0166397.s004.tif]

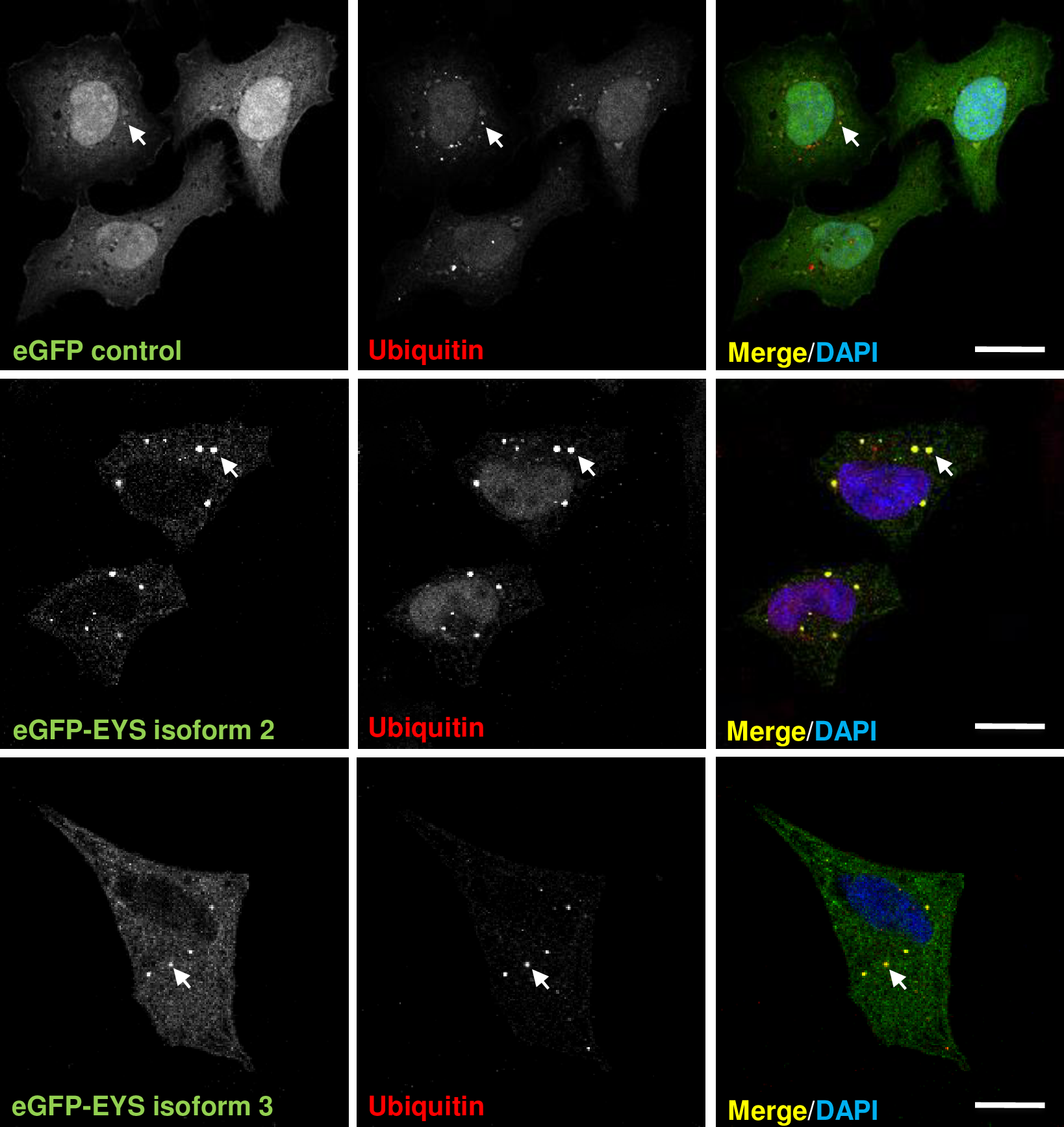

Supplement: S5 Fig — The white arrows indicate the speckles of protein going through the ubiquitin-proteasomal protein clearance system of the cell. Cell nuclei are labelled with DAPI (blue). Scale bars: 10 μm. (TIF) [file pone.0166397.s005.tif]
