# Supplementary material for: EYS Is a Protein Associated with the Ciliary Axoneme in Rods and Cones
Source: PLoS One. 2016 Nov 15;11(11):e0166397. doi: 10.1371/journal.pone.0166397 (PMC5112921; doi:10.1371/journal.pone.0166397)
Supplement: S1 Table — (DOC) [file pone.0166397.s006.doc]

**S1 Table. A summary of primer sequences used for RT-PCR analysis of transcript levels of EYS isoforms in the human retina and Y79 cell line**

| **Primer** | **Sequence (5'->3')** |
| --- | --- |
| EYS isoform 2 Forward | CCCGAAAATGACTGACAAATC |
| EYS isoform 2 Reverse | GCCACTAAATCAAACTTATATTC |
| EYS isoform 3 Forward | CCCGAAAATGACTGACAAATC |
| EYS isoform 3 Reverse | CTGCTCAAATGATACATAAATACC |
| EYS isoform 4, 42nd exon Froward | CCATCTAGATCCAGGTAGCC |
| EYS isoform 4, 42nd exon Reverse | CCATCTAGATCCAGGTAGCC |
| HPRT Froward | GGGACATAAAAGTAATTGGTG |
| HPRT Reverse | GCGACCTTGACCATCTTTGG |
